# Comparison of the Explantation Rate of Poly Implant Prothèse, Allergan, and Pérouse Silicone Breast Implants within the First Four Years after Reconstructive Surgery before the Poly Implant Prothèse Alert by the French Regulatory Authority

**DOI:** 10.1155/2015/519497

**Published:** 2015-10-12

**Authors:** Alexandre Leduey, Chafika Mazouni, Nicolas Leymarie, Heba Alkhashnam, Benjamin Sarfati, Jean-Rémi Garbay, Amélie Gaudin, Frédéric Kolb, Françoise Rimareix

**Affiliations:** ^1^Department of Breast and Plastic Surgery, Gustave Roussy, 94805 Villejuif, France; ^2^Department of Pharmacology, Gustave Roussy, 94805 Villejuif, France

## Abstract

*Background*. In March 2010, ANSM (Agence Nationale de Sécurité du Medicament), the French Medical Regulatory Authority, withdrew Poly Implant Prothèse (PIP) breast implants from the market due to the use of non-medical-grade silicone gel. The aim of this study was to compare the removal rate (and reasons thereof) of breast implants produced by different manufacturers before the ANSM alert. *Materials and Methods*. From October 2006 to January 2010, 652 women received 944 implants after breast cancer surgery at the Gustave Roussy Comprehensive Cancer Center, Paris (France). The complications and removal rates of the different implant brands used (PIP, Allergan, and Pérouse) were evaluated and compared. *Results*. PIP implants represented 50.6% of the used implants, Allergan 33.4%, and Pérouse 16%. The main reasons for implant removal were patient dissatisfaction due to aesthetic problems (43.2%), infection (22.2%), and capsular contracture (13.6%). Two years after implantation, 82% of Pérouse implants, 79% of PIP, and 79% of Allergan were still *in situ*. There was no difference in removal rate among implant brands. *Conclusion*. Before the ANSM alert concerning the higher rupture rate of PIP breast implants, our implant removal rate did not predict PIP implant failure related to the use of nonapproved silicone gel.

## 1. Introduction

Immediate or secondary breast reconstruction can be performed using a breast implant alone or associated with a flap. Breast reconstruction with implants is almost the first choice for many women after breast cancer surgery because it allows rapid recovery, greater ability to change the breast volume, and avoidance of donor site morbidity [1, 2].

Breast implants have been developed and used for breast reconstruction for more than 40 years. Breast implants are manufactured as pliable silicone elastomer shells that are filled with silicone gel or saline solution. Silicone gel mimics the natural breast tissue more closely. Manufacturers have improved the implant safety and created many new models over the years. The last generation of implants has a multilayer shell with a barrier layer and is filled with thick silicone gel [3]. Many studies on the rate of complications, especially the rupture of silicone breast implants, during aesthetic or reconstructive surgery have demonstrated that silicone implants are safe and effective [4–7].

In early 2010, ANSM (Agence Nationale de Securité du Médicament), the French Medical Regulatory Authority, suspended the marketing, distribution, and use of silicone gel-filled breast implants produced by Poly Implant Prothèse (PIP) due to the use of a nonapproved filler material. Moreover, these breast implants did not have any elastomeric shell and the gel could more easily ooze through an intact implant shell and cause local irritation [8–11].

ANSM recommendation to the medical community was to monitor women with a PIP implant by ultrasound scan every six months to identify any implant rupture. At that time, systematic removal of PIP breast implants was not endorsed in the absence of conclusive scientific evidence concerning the gel toxicity [12]. However, on December 23, 2011, the French Ministry of Health recommended prophylactic surgery to remove PIP implants.

In the interval between the ANSM alert and the final recommendation about prophylactic surgery, the management of patients with PIP implants was left to the surgeon's discretion. Due to the growing media and patients' anxiety [13], the surgical team at the Gustave Roussy Comprehensive Cancer Center, Paris (France), decided to prophylactically remove all PIP breast implants.

A previously published study on systematic PIP implant removal after the ANSM alert reported high rates of breast implant leakage, but no rupture, with an average PIP breast implant lifespan of 21 months [14]. Another retrospective study to determine the rupture and complication rates of PIP breast implants found a high rate of implant rupture (7.7%) associated with periprosthetic effusion in 44% of these patients [15]. However, no study has demonstrated that the rate of PIP breast implant complications is higher than that of other brands during the early years after implantation. Here, we evaluated and compared the rate of and reasons for removal of PIP, Allergan, and Pérouse breast implants (the three main breast implants brands used in our center) before the ANSM alert to determine whether any significant clinical events could have predicted the corporate fraud.

## 2. Patients and Methods

For this study, we retrospectively evaluated the data concerning all women who underwent breast reconstruction with breast implants at the Gustave Roussy Comprehensive Cancer Center (Paris, France) from 2006 to 2010. During this study period, PIP was the most frequently used breast implant.

Information on the implant brand, manufacturer, lot number, and lifespan was retrieved, after excluding all patients who had expander-based breast reconstruction and those who received Sebbin breast implants, which represented only 1% of the implants and were samples given by the manufacturer to be tested. We also identified the implantation date and all breast implant explantations carried out during the study period.

The breast implant lifespan by brand was determined before the ANSM alert and the prophylactic removal of PIP implants.

We also identified and recorded the causes leading to additional surgery and implant replacement. Causes of premature implant removal included, but were not limited to, rupture, capsular contracture, pain, distortion, rippling and wrinkling, infection, delayed healing with or without implant exposure, and patient dissatisfaction.

Quantitative data were compared using Pearson's chi-square and Fischer's exact tests. Factors predictive of implant removal were analyzed using a logistic regression model and presented as odds ratios (OR) with their 95% confidence interval (95% CI). The lifespan of the different implant brands was compared using the Kaplan-Meier method. A *p* value < 0.5 was considered statistically significant.

## 3. Results

During the study period (between 2006 and 2010), 652 patients received a total of 944 breast implants (14% of patients had bilateral breast reconstruction) following breast cancer surgery. The most used breast implants were PIP implants (50.6%; *n* = 478), especially because of their asymmetric shape, followed by Allergan silicone gel implants (33.4%; *n* = 315) and then Pérouse silicone gel implants (16%; *n* = 151). These implant-based breast reconstructions were carried out by six surgeons.

Immediate breast reconstruction was the main indication for breast implant (53%), followed by secondary breast reconstruction (29%), contralateral breast augmentation (13%), and implant exchange to improve breast shape (4%). All implants were textured, 91% were asymmetric, and only 9% were round. All implants were placed in a subpectoral position.

**Figure 1:**
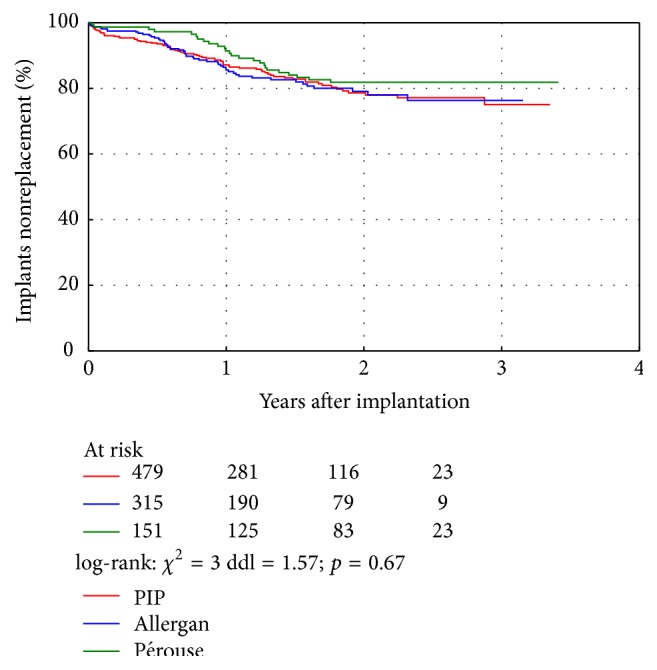
Breast implant longevity.

Two years after implantation, 82% of Pérouse implants and 79% of PIP and Allergan implants were still* in situ* (Figure 1). There was no difference between the brands in terms of implant lifespan (*p* = 0.67).

Overall, 158 of the 944 breast implants (16.7%) were removed: 81 were PIP implants (17% of all PIP implants), 52 were Allergan implants (16.5%), and 25 were Pérouse implants (16.5%). The implant removal rate was not statistically different among the three brands (*p* = 0.43). The average time to explantation was 299 days (4–1050 days).

During the study period (before the ANSM alert), PIP implants were removed for the following reasons (Table 1): unsatisfactory aesthetic results that required implant replacement (*n* = 35 patients), infection that required immediately further surgery (*n* = 18 patients), implant exposure (*n* = 5 patients), suspected rupture that was confirmed intraoperatively (*n* = 4 patients), capsular contraction (*n* = 11 patients), migration or rotation of the implant (*n* = 2 patients), or recurrent cancer (*n* = 2). Comparison of the causes that led to implant replacement did not highlight any significant difference between PIP and Allergan/Pérouse implants (Table 1), although the infection rate tended to be higher (*p* = 0.054) in the PIP group.

## 4. Discussion

Our study compared the rate and causes of PIP implant replacement with that of other implant brands used in our center (Allergan and Pérouse) during the first years after implantation. Our objective was to determine whether some undesirable events could have alerted surgeons before the ANSM alert about the higher rupture rate among PIP breast implants on March 29, 2010 [8].

Since the introduction of the first breast implants in the 1960s, their design and manufacturing have changed radically with the arrival of cohesive gels, thick prosthetic membranes, textured surfaces, and improved quality control. The current breast implants offer better safety with less implant rotation and less capsular contracture [6, 16]. This qualitative improvement was the reason for their return to the French market in 2001 after being banned for several years. Cohesive gel implants are considered to be the undisputed gold standard for breast reconstruction. Breast implant factories are now regularly inspected and adverse events must be declared to the ANSM.

The abnormal increase in the rupture rate of PIP implants led to more frequent inspections of the PIP implant factories. This revealed serious shortcomings in the manufacturing of these products. The silicone gel used was not of medical grade with a low polymerization rate and many short chain residues that leak more easily through the implant shell. This side effect was even more a cause for concern because it reduces the efficacy of the implant shell by increasing its porosity [9, 11].

Between 2006 and 2010 most of the implants fitted in our surgery department were PIP products (50.6% of patients with breast reconstruction had a PIP implant fitted). Before a medical device is available in our institution, it has to be referenced by the French Federation of Cancer Centers (UNICANCER), based on the evaluation of its safety and technical advantages compared to the already used material. In 2006, PIP implants were introduced into our department because they offered a larger base width and an anatomical shape that gave a more natural breast for reconstruction, compared to other brands [16]. Therefore, we began to use them frequently due to their advantages in terms of shape and final aesthetic results.

The incidence of implant failure is generally low [17, 18]. In our population, 16.7% of patients needed an implant replacement before four years. We did not find significant differences between the rates of PIP removal and that of the other brands. Interestingly, no report about an abnormal removal rate of PIP implants was published before the ANSM alert. Moreover, the major causes of breast implant removal were not related to the gel mechanical and viscoelastic properties, but to aesthetic revision, capsular contracture, and infection, like for the other brands. The similar four-year reoperation rate due to aesthetic problems for the three brands indicates that the mechanical and viscoelastic properties of the gel used for the PIP implants did not influence the aesthetic result. However, previous studies reported the anomalous early tendency of PIP implants to disintegrate shortly after implantation and the inflammatory response caused by the leakage of silicone gel [19–22]. Our study highlighted a trend towards a higher infection rate with PIP implants (*p* = 0.054) for which we have no explanation. Bacterial culture was not carried out in all cases of infection and antibiotics were administered in the presence of clinical signs of inflammatory reactions. For all implants, the body naturally forms more or less tight capsules that could give rise to capsular contracture, leading to their removal. Most capsular contractures are observed during the early years after implant placement. Our results suggest that the rate of capsular contractures is not affected by the biomedical quality of the silicone gel, but more by the choice of the pocket and the implant surface [23].

Breast implant removal due to implant rupture within the first four years after surgery concerned 4.9% of the patients with a PIP implant compared to 0% for the other brands. In 2006, Hedén et al. reported 6% (*n* = 199) of ruptured implants during a mean follow-up period of 10.9 years and only 0.3% of ruptures during the first five years [24]. The implant rupture rate ranges from 0.3 to 77% in the literature [24–30] due to the implant heterogeneity and manufacturing changes during recent years. The risk is cumulative and is estimated to be approximately 6% per year during the first five years following the primary implant surgery [29]. Our failure rate was low: 0.8% at four years for all implants. The rupture rate of PIP silicone implants (4.9%) was not significantly higher compared to that of other brands (only four PIP implant ruptures). This rate is low and was based on removal due to clinical suspicion of implant rupture before the systematic monitoring/explantation of PIP breast implant after the ANSM alert. Therefore, the rupture rate in our series of patients with breast cancer was probably underestimated, compared to the rupture rate (16.1%; 4130/25644 implants) after systematic explantation of PIP implants reported by ANSM [31]. Even higher PIP rupture rates (30.9% in 2000 and 35.1% in 2001) were observed by Maijers and Niessen [32]. Moreover, the ANSM reported an overall rate of implant failures (rupture, gel bleeding, and capsule contraction) or other adverse events of 21% after preventive explantation.

The PIP implant biodurability was related to the implantation year, with a median time to rupture of 10.5 years in 2000 and 5.8 years in 2005, showing that PIP implants were becoming less durable [33]. Recent studies suggest that the use of medically unapproved silicone gel in the PIP implants did not contribute to their rupture [32], which was mainly caused by the shell weakness or shell failure due to different variables, including variable shell thickness, the nature of shell texturing, sharp corners, and identification marks [11].

We did not evaluate the rupture rate after the ANSM alert in 2010. Although our study reveals that the rupture rate of PIP implants was not significantly different from that of other brands, we must not forget that the silicone gel used was not approved by the regulatory medical authority. We therefore agree with the recommendation that PIP implants should be removed prophylactically. Besides the physical danger of these implants, there is also an important psychological factor, especially in patients who had been treated for breast cancer and who should be spared additional worries.

## 5. Conclusion

Before the discovery that medically unapproved silicone gel was used in PIP breast implants, the reasons for implant removal would not have allowed us to unmask the manufacturer's fraud. Indeed, implant rupture was not significantly more frequent with PIP than with Allergan or Pérouse breast implants.

## Figures and Tables

**Table 1:** Reasons for breast implant removal.

Complications	PIP (81)	Allergan/Pérouse (77)	OR	95% CI	*p*
Infection	18 (22.2)	8 (10.4)	2.45	[0.9; 6.9]	0.054
Exposure	5 (6.2)	5 (6.5)	1.05	[0.2; 4.7]	1
Rupture	4 (4.9)	0 (0)	—	[0.63; —]	0.12
Capsular contracture	11 (13.6)	17 (22.1)	0.59	[0.2; 1.5]	0.3
Aesthetic reoperation	35 (43.2)	37 (48.1)	1.05	[0.5; 2.1]	1
Migration	2 (2.5)	6 (7.8)	0.3	[0.03; 1.8]	0.16
Cancer recurrence	2 (2.5)	1 (1.3)	1.9	[0.1; 114.9]	1
Others	4 (4.9)	3 (3.9)	1.2	[0.2; 9.03]	1
Number (%)	81 (100)	77 (100)	0.97	[0.7; 1.4]	0.93

OR: odds ratio; CI: confidence interval.
